# Supplementary figures and images for: Spatial cluster analysis of Plasmodium vivax and P. malariae exposure using serological data among Haitian school children sampled between 2014 and 2016
Source: PLoS Negl Trop Dis. 2022 Jan 5;16(1):e0010049. doi: 10.1371/journal.pntd.0010049 (PMC8765618; doi:10.1371/journal.pntd.0010049)

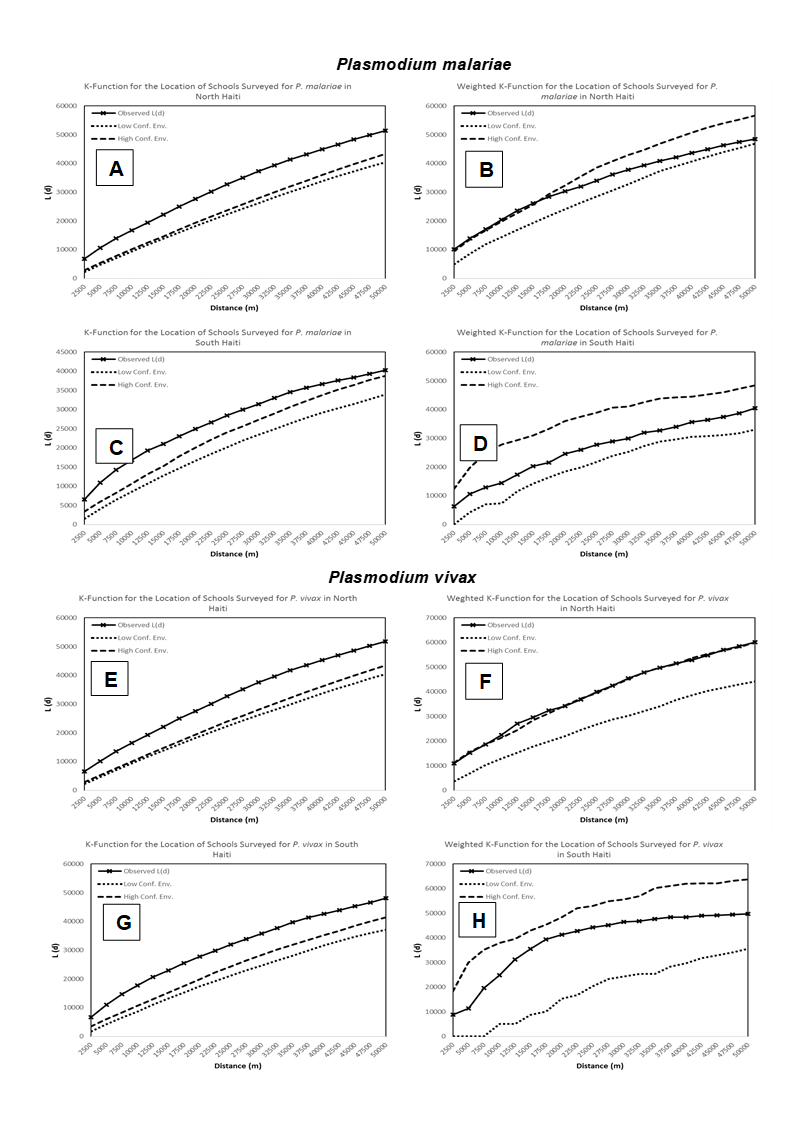

Supplement: S1 Fig — K-functions and weighted K-functions detailing spatial randomness of schools surveyed for IgG antibodies against P. malariae (A-D) and P. vivax (E-H) in Haiti. Spatial scan area was divided into northern and southern regions in order to avoid empty space bias caused by lack of sampling in the Ouest department. For unweighted fields, confidence envelopes are generated by distributing points randomly in the study area and calculating k-values for 999 permutations. For each distance, the highest and lowest deviation from the expected K-value construct the envelope. The same methods are used for weighted fields with the exception that only the weighted values are randomly distributed to generate confidence envelopes; point locations remain fixed. (TIF) [file pntd.0010049.s001.tif]

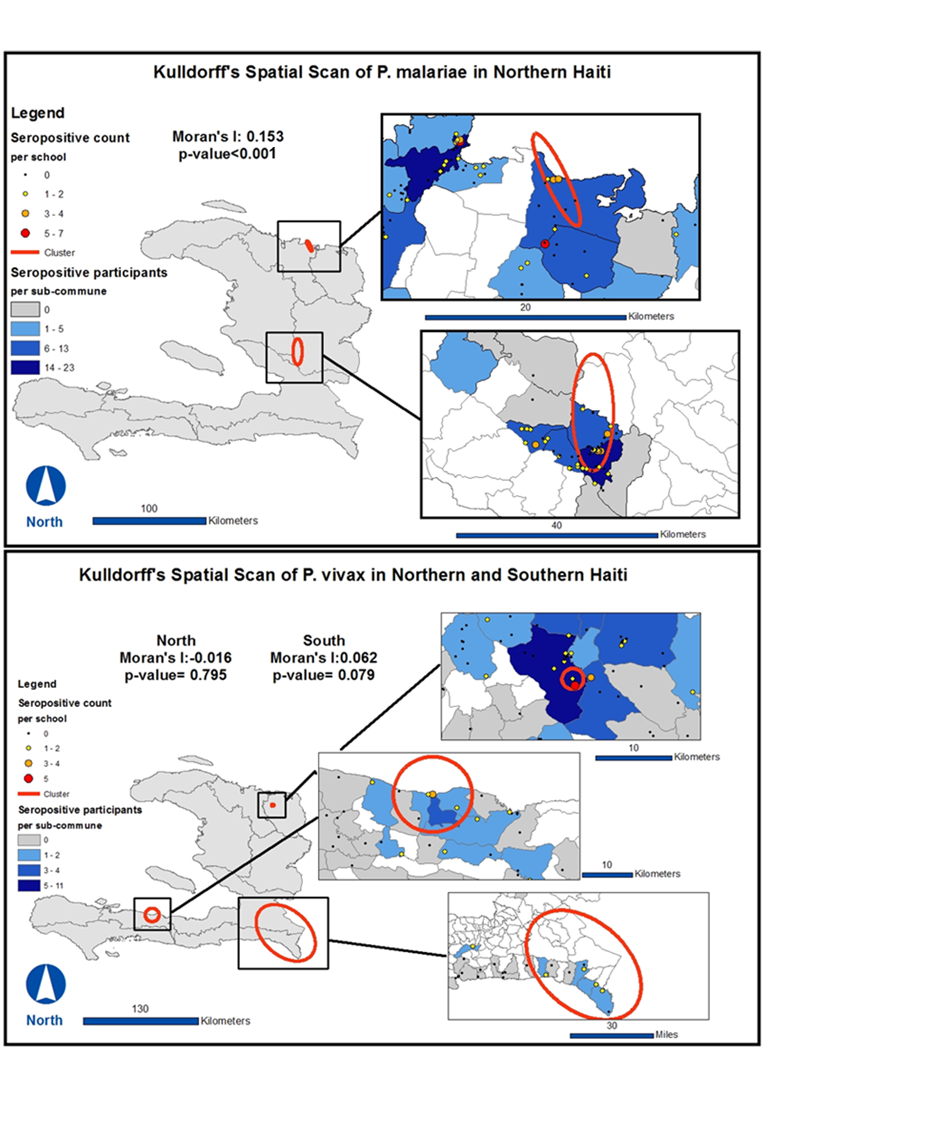

Supplement: S2 Fig — Kulldorff’s Spatial Scan for seropositive counts for P. malariae (top panel) and P. vivax (lower panel) as divided into the northern and southern sections of the country. Ellipses denote cluster borders, and shading of sub-communes indicate number of seropositive children enrolled during the TAS. Base map for administrative boundaries found at: https://www.geoboundaries.org/index.html#getdata. (TIF) [file pntd.0010049.s002.tif]

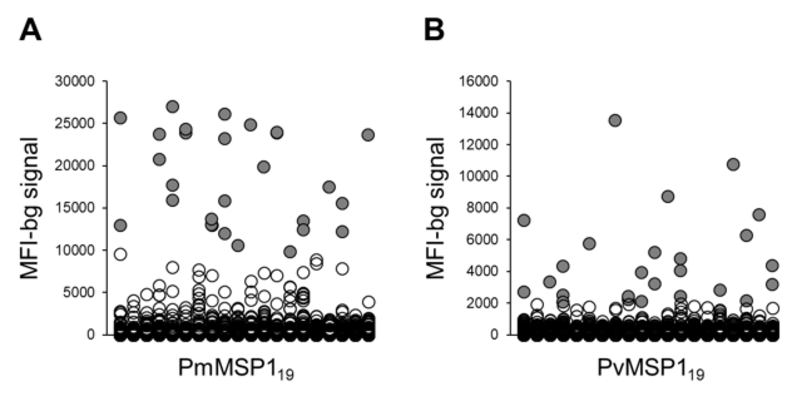

Supplement: S3 Fig — Selection of samples with high IgG assay signal to PmMSP119 (A) and PvMSP119 (B) for further laboratory tests. Samples providing assay signals in the grey circles were selected. (TIF) [file pntd.0010049.s003.tif]
