# Supplementary material for: Spatial cluster analysis of Plasmodium vivax and P. malariae exposure using serological data among Haitian school children sampled between 2014 and 2016
Source: PLoS Negl Trop Dis. 2022 Jan 5;16(1):e0010049. doi: 10.1371/journal.pntd.0010049 (PMC8765618; doi:10.1371/journal.pntd.0010049)
Supplement: Attachment — Submitted filename: Oviedo_etal_Response_to_Reviewers.docx [file pntd.0010049.s005.docx]

**Oviedo, et al, Response to Reviewers**

**PNTD-D-21-00655**

The authors thank the reviewers and editor for practical and fair review points in order to improve this submission. We have attempted to comprehensively answer all comments below.

<b>Key Review Criteria Required for Acceptance?</b></br></br> As you describe the new analyses required for acceptance, please consider the following:</br></br>

<b>Methods</b></br></br>

-Are the objectives of the study clearly articulated with a clear testable hypothesis stated?</br> -Is the study design appropriate to address the stated objectives?</br> -Is the population clearly described and appropriate for the hypothesis being tested?</br> -Is the sample size sufficient to ensure adequate power to address the hypothesis being tested?</br> -Were correct statistical analysis used to support conclusions?</br> -Are there concerns about ethical or regulatory requirements being met?</br></br>

Reviewer #1: See all comments below

Reviewer #2: - Provide a clearer explanation on the differences between each of the three spatial/cluster analyses used. Each of the sections in the results talk about “clustering” but its hard to tell how the interpretations between the three methods fit together.

- Provide details in the statistical analysis section on the use of Bayesian logistic model, and it is not clear in the results as to why this was performed and why the OR estimates were inconsistent for NDVI and LST. This finding seems very strange for univariate multilevel logistic models – what were the priors, how influential? Also for the multilevel logistic model more details should be provided on the random effects – e.g. for school clustering.

These Bayesian models and environmental variables have been removed from body and figures. Figure numbers have been updated.

Reviewer #3: The authors clearly stated their objective to establish seroprevalence of malaria species in Haiti. They used a young age group collected at schools to do this in order to look at recent infections and were able to establish appropriately large sample sizes (21,719 students from 680 schools). They used appropriate statistics to test for the degree of spatial aggregation of the schools, and autocorrelation of seropositivity counts, followed by Kulldorff’s Bernoulii scan to identify significant hotspots.

--------------------

<b>Results</b></br></br>

-Does the analysis presented match the analysis plan?</br> -Are the results clearly and completely presented?</br> -Are the figures (Tables, Images) of sufficient quality for clarity?</br></br>

Reviewer #1: See all comments below

Reviewer #2: - Figure 1: need to have higher resolution on these figures.

- Table 1 – 2: It should be made clearer in Tables 1 and 2 that the OR presented for age, elevation, NDVI and LDT are for these as continuous covariates (also did the authors investigate if the association between these continuous covariates and the log odds of seropositivity was linear, as no details are provided in the statistical analysis section), and the units should be given in the variable column for age. Also the reference group for gender should be specified. ‘Variable’ is not spelt correctly in Table.

These models and environmental variables have been removed and “variable” has been spelt correctly.

- Line 343 – 347: the 95% CI should be provided in brackets next to the OR estimate, and the exact p-value instead of a categorised p-value. (Should be “<” instead of “>” on line 343 – 344 if you decide to keep these p-values in text).

These models have been removed.

- Line 363: change “detail” to “correspond to”.

This has been updated.

- Figure 2: consider giving one common legend for this figure rather than splitting between the two plots.

We thank the reviewer for this suggestion. Due to the shape of Haiti, we would be unable to include all the information in one condensed legend, but we feel the reader understands the legend designations apply to both panels.

- Figure S2: explain the acronyms shown in the figures (L(d), Low Conf. Env., High Conf. Env.) in the figure legend.

Further details are now included in the figure description.

- Line 389 - 414: give actual p-value rather than categorising as >0.05 and <0.01. Replace by 95% CI if this is available.

The authors thank the reviewer for this suggestion, but feel that categorizing the p values into commonly used categories (p>0.05, p<0.01, etc.) helps the reader to more easily interpret the magnitude of statistical significance.

- Table 3: include information on method used in the legend, for example: “by Kulldorff’s scan statistics.” Please also provide confidence intervals for the RR estimates in the table and in text if available.

Table description has been updated to include methods. Unfortunately SatScan (Version 9.6) does not provide confidence intervals for RR estimates.

- Line 426: can remove “as described in Methods”.

This has been removed.

- Line 426: remove the ’ at the end of children’s.

This has been removed.

Reviewer #3: The results establish the geographical distribution of cases of various malaria species in Haiti as the authors intended. Drawing figure 3 as a superimposition over cases, as was done in S3, may help readers see the pattern more effectively.

The authors thank the reviewer for this suggestion, and this had been attempted in a previous version but the resulting figure was too crowded to be able to make sense of.

--------------------

<b>Conclusions</b></br></br>

-Are the conclusions supported by the data presented?</br> -Are the limitations of analysis clearly described?</br> -Do the authors discuss how these data can be helpful to advance our understanding of the topic under study?</br> -Is public health relevance addressed?</br></br>

Reviewer #1: See all comments below

Reviewer #2: - The discussion could be made stronger by having a summary of the key findings, which address the research questions detailed in the introduction, earlier in the discussion section (at least in the first paragraph).

The authors thank the reviewer for this suggestion, and have modified the Discussion to include these points raised.

- Line 462: consider removing the word “vanishingly”, small is enough.

This has been removed

- Line 512 – 513: “autocorrelation measures clearly suggest that high seropositive counts do not neighbor other high counts” – but in the results section autocorrelation results showed clustering of high P. malariae seropositivity in the north?

The authors thank the reviewer for pointing this out. The sentence was updated to more accurately outline the results.

- Include in the discussion section recommendations on how the detection of hotspots using Kulldorff’s scan statistic (Table 3) could be used – do the authors recommend anything based on these hotspot results?

The authors thank the reviewer for this comment, and have now added two additional sentences to the Discussion: “Practically, detection of hotspots of seropositivity for non-dominant malaria species could provide rationale for follow-up studies in these specific areas in attempt to obtain more granular spatial estimates for tangible programmatic purposes such as targeted mass-drug administration (MDA) in smaller populations. Additionally, health facilities within hotspot areas could be put on notice and given appropriate diagnostic tools to take into account non-dominant species when diagnosing a patient with ‘malaria-like symptoms’.”

Reviewer #3: Conclusions are generally well supported by the data presented and limitations are mostly well addressed. One exception would be the exclusion of P. ovale within the study is not mentioned when discussing relative species prevalence.

The authors thank the reviewer for this suggestion, but P. ovale transmission has never been reported in the New World.

--------------------

<b>Editorial and Data Presentation Modifications?</b></br><br/> Use this section for editorial suggestions as well as relatively minor modifications of existing data that would enhance clarity. If the only modifications needed are minor and/or editorial, you may wish to recommend “Minor Revision” or “Accept”.

Reviewer #1: See all comments below

Reviewer #2: - Line 1-3: consider including something about seroprevalence or serological data in the title.

This has been included

- Line 28 – 29: include study aims at the end of the background section of the abstract.

The authors thank the reviewer for this suggestion, but currently have already maxed out the word limit for the Abstract. We feel the text already included in the Abstract conclusions/significance section point to what we aimed to do with this study.

- Line 33: can remove the word “later” for simplicity.

This has been removed

- Line 56 – 57: consider rewording this sentence to: “P. falciparum is the dominant malaria species worldwide and is often the primary, or only, focus of malaria surveys.”

Thank you for this suggestion, and we have replaced this sentence as suggested.

- Line 58 and 62 – change “populace” to “population”.

This has been changed

- Line 70 – 71: consider rewording to “Identification of Plasmodium reservoirs in low transmission settings rely on conventional diagnostics (microscopy and RDT) which underestimate the true incidence and prevalence.”

This has been changed

- Line 72 – 73: antibody-based assays are a type of diagnostic. We would suggest rewording “When compared to diagnostic tests” to “When compared to microscopy and RDTs”.

This has been changed

- Line 73: suggest removing “more powerful indicator for detecting active infection…” in place of “more powerful method for detection of active infection…”.

This has been changed

- Line 74 – 75: consider simplifying the last part of this sentence to “expanding the time window for diagnosis.”

This has been changed

- Line 88: consider changing “reduction efforts” to “elimination efforts”.

This has been changed

- Line 100 – 104: an overview of what is done in the study is given but should also have clear aims stated.

The authors agree with this suggestion and have added an additional sentence to the end of the Introduction section: “These analyses aim to provide rationale for utilizing IgG data to determine spatial transmission patterns for very low endemic malaria species in an area.”

- Line 100: remove the word “occurring”.

This has been changed

Reviewer #3: In figure S3, in the top panel, I suggest the P-value be written using scientific notation so that it is not P < 0.000

This has been changed to “p-value<0.001”

Line 132: extra parenthesis at the end of the sentence

This has been removed

Line 142: missing space

This has been added

Line 379: S1F figure should likely be S2F

This has been corrected

Line 399: Combine table & figure references to be more consistent with previous reference formats

This has been corrected

Line 442-444: Sentence lacks appropriate subject

This has been corrected

--------------------

<b>Summary and General Comments</b></br></br> Use this section to provide overall comments, discuss strengths/weaknesses of the study, novelty, significance, general execution and scholarship. You may also include additional comments for the author, including concerns about dual publication, research ethics, or publication ethics. If requesting major revision, please articulate the new experiments that are needed.

Reviewer #1: Oviedo and colleagues describe results from a large-scale sero-survey of >20,000 school children in Haiti, with a particular focus on P. malariae and P. vivax. They identified low-levels of sero-positivity against both P. malariae and P. vivax MSP1-19, with evidence of some (independent) spatial clusters. In a subset of samples tested, they found no evidence of active Plasmodium infections. The article is well written, logical and with sound data analysis. I have only minor comments that I as a reader would find helpful for the authors to address.

1. Cross-reactivity between Pf, Pm, Pv MSP1-19

The authors seem confident that there would be minimal cross-reactive antibody responses against the various MSP1-19 proteins tested from the three species, based on past literature and their results in Figure 1.

a. I suggest in the methods that the authors do detail the sequence identity between the Pf-Pv Pf-Pm and Pv-Pm MSP1-19 protein sequences (I believe it is moderate at least for Pv-Pm around 56%).

We agree with this suggestion, and have added a line in Methods: “Overall sequence consensus among these three recombinant 19kD antigens was low at 34.4%.”

b. In the results description for Figure 1, it would be helpful to quantify how many children had a positive signal to more than one MSP1-19 antigen out of the total children positive with any positive signal. This is detailed around lines 318/319 for Pm and Pv but doesn’t include Pf, and clearly from Figure 1 there are quite a few positive antibody signals for Pf.

The authors thank the reviewer for this suggestion, but the focus for this study was only for seropositivity to Pm and Pv antigens, and the Pf data will be more extensively presented elsewhere. The inclusion of Pf data in Fig 1 was primarily to indicate specificity of the assay we were using.

2. Results for Pf MSP1-19

I appreciate that the authors have focused their results on Pv and Pm given the prevalence/transmission of these species in recent years in Haiti remains unknown. However they did measure antibody against PfMSP1-19 so I think it is worth at least documenting/quantifying the proportion of the children seropositive to Pf-MSP1-19 even if the subsequent detailed analysis is only on Pm and Pv. This could be added to the text description for Figure 1, and added into Line 459 in the discussion.

The authors agree the Pf serology data is also relevant in this population, but do not want to detract away from the focus of this current study, which is only for Pm and Pv serology. As this is the dominant Plasmodium species in Haiti, a more extensive report on Pf serological findings will be forthcoming.

3. Remaining minor points

a. Line 75 seems to be an aberrant comma

This has been removed.

b. Line 81 define MSP1 for first use

This has been defined.

c. Line 142 missing space between C and until

This has been corrected.

d. Line 164 text says City, STATE – needs adding in

This has been added.

e. Lines 155, 173, 204 please provide more details. What is the positive control? How many negative controls were run to generate the seropositivity cut-off?

This information has been added.

f. Would Figure 1B be clearer on a log scale like Figure 1A?

The authors thank the reviewer for this suggestion, but feel this format on the non-transformed scale is more important for emphasizing the moderate and higher assay signals - which are the seropositive signals.

g. Line could be helpful to give more context – latest % prevalence estimate? Or # cases per year?

Unfortunately the reviewer doesn’t provide a line number here, so the authors are unsure of the section being referred to.

h. Line 456 would antibody longevity be expected to be similar between adults and children?

Duration of IgG positivity to MSP-1-19kD antigens has been modeled to be many years for both adult and child populations.

i. In the discussion, the authors could comment on their use of binary seropositivty data, would added value be gained from using the actual antibody magnitude?

The authors thank the reviewer for this suggestion. However, in this very low Pv and Pm prevalence setting (<2% for either), there would have been no real additional value in continuous IgG data (or approximating titers).

j. For the discussion, can the authors comment on whether there are any geographic or human features that associate with the clusters? Like village boundaries, or rivers/mountains/roads etc? And perhaps why Cluster D is so large (is this an effect of less schools being tested in that region)?

The authors thank the reviewer for this suggestion, but have addressed this point regarding cluster size in Discussion Line 507: “any clustering of seropositive children could potentially be dependent on school location and therefore be over-estimated due to the aggregation of school sample locations”. Cluster size will be a factor of presence of seropositive children as well as density of schools sampled in a spatial area.

k. Do the authors think it is worthwhile doing their PCR assays/antigen assays on the whole sample set in the future to search for active Pm and Pv infections or would that be waste of time/resources?

If attempting to investigate the presence of active infections in a very low endemic setting such as this (for Pv/Pm in Haiti), it would not be of much value epidemiologically to run antigen or DNA detection for such a large number of samples. Perhaps the investigators would catch a few positives throughout the country, but it would be impossible to use this data for any spatial predictions or other statistical efforts.

I selected "no" for is all the underlying data available as it was not included as a Supplement so I personally could not access it. The corresponding author does say it is available upon request so the Editor can determine whether this is appropriate or not.

Reviewer #2: The manuscript “Spatial cluster analysis of Plasmodium vivax and Plasmodium malariae exposure among Haitian school children sampled between 2014 and 2016” presents an interesting analysis of a very important issue. As malaria declines it is essential that we understand the changing dynamics of transmission. This study will provide useful information to those working in malaria elimination in Haiti and those who are interested in how serological data can supplement existing diagnostic methods.

The comments provided here recommend further improvements to the manuscript.

Reviewer #3: The study by Oviedo et al provides a serosurvey of P. malariae, P. vivax and P. falciparum in Haiti between 2014 and 2016. The study focuses on young children who have just a few years of potential exposure so the data presented is valuable in its ability to inform scientists on the degree to which these species have been endemic in Haiti over the last few years. The use of spatial clustering methods established areas where each species is likely to be most endemic and may be useful in elimination efforts. The methodology seems appropriate for the conclusions. While there are some limitations that result from sampling bias, the authors address this multiple times in their results and discussion. I recommend this manuscript be accepted, though I have some minor recommendations:

Sources 5 and 23 both appear to reveal plausibility for detection of P. ovale, and the authors mention that the last surveys in this area were in the 60s. Further, they assert that results may indicate P. malariae is the second most common on lines 470-472 without having data for P. ovale available. Authors should discuss why they chose to not include P. ovale and to consider this in their assertions.

The authors thank the reviewer for this suggestion, but inclusion of a Po antigen is not warranted as Po transmission has never been reported in the New World.

Authors should comment on why they chose not to adjust for collection or population numbers.

The authors are not quite sure what the reviewer is recommending here. The analyses presented take into account the fact that some areas of the country are under-sampled (or not sampled at all).

I also noted the following minor writing errors:

Line 132: extra parenthesis at the end of the sentence

This has been corrected.

Line 142: missing space

This has been corrected.

Line 379: S1F figure should likely be S2F

This has been corrected.

Line 399: Combine table & figure references to be more consistent with previous reference formats

This has been changed.

Line 442-444: Sentence lacks appropriate subject

This sentence has been corrected.

Figure S3, in the top panel, I suggest the P-value be written using scientific notation so that it is not P < 0.000

This has been changed to now read “p<0.001”

Deputy editor:

The statistical analysis appears a little weak. The methods chosen are not well justified, and there is a heavy reliance on scan statistics which while simple and easy to run can only say whether there is a significantly higher prevalence in an area but not how much higher (which would be useful for control activities). In figure 3, the hotspots for P vivax are very large, but the prevalence is unlikely to be uniformly high across this area, there are relatively few cases and it does not correspond well visually to Fig 2. (A prevalence map may be an option, although would require more sophisticated statistical methods). I suggest reducing the emphasis on the cluster analysis, and removing the section on spatial randomness since malaria is well known to be heterogenous on many scales. The size of the clusters does not seem to be justified - ideally it would bear relevance to control activities or whatever the information is used for. In Fig S3, the elipses cover areas with no data and so the shape seems rather inflexible. Whether you choose scan statistics or other analysis, the methods should be justified and limitations mentioned in the discussion.

For the univariate logistic regression models, clustering within schools does not look as if it has been taken into account, and the choice of univariate is neither obvious nor justified. A statistician would be able to advise on this.

The authors thank the reviewer for this suggestion. A more detailed limitations section was included to address these points, and discuss practical implications and usage for these spatial predictions. Overall, as the transmission of falciparum malaria (or a country with more prevalent vivax malaria) will ultimately drive programmatic decisions, the authors do not attempt to propose that large spatial areas of seropositivity to non-dominant Plasmodium sp. is going to drive decision making, but rather attempt to show that this data is uncovering the truth to neglected malaria species in Haiti. The ramifications would be applicable to numerous other global settings which are only (currently) collecting data on falciparum malaria but seek to eliminate all Plasmodium parasites from their populace. We have added additional text to the Discussion in order to point to potential tangible steps that could be taken with these spatial outputs.

In addition, regression models have been removed to satisfy the reviewers’ and Editor’s concerns.
