# Supplementary material for: Spatial cluster analysis of Plasmodium vivax and P. malariae exposure using serological data among Haitian school children sampled between 2014 and 2016
Source: PLoS Negl Trop Dis. 2022 Jan 5;16(1):e0010049. doi: 10.1371/journal.pntd.0010049 (PMC8765618; doi:10.1371/journal.pntd.0010049)
Supplement: Attachment — Submitted filename: Oviedo_etal_Resubmission2_Response to Reviewers.docx [file pntd.0010049.s006.docx]

**Oviedo, et al, Resubmission Response to Reviewers**

**PNTD-D-21-00655R1**

The authors thank the reviewers and editor for additional helpful comments to create a better product. We have attempted to comprehensively answer all comments below.

**Reviewer #1**:

In all Tables, needs to have the Plasmodium species in italics.

- The authors regret this oversight and have corrected this error.

In Table 1 and Table 2, the "a" after schools could be in superscript then it would look less like a typographical error.

- The authors have changed this.

For clarity, I would suggest noting in the results or discussion that analyses and presentation of the Pf data will be in an upcoming publication.

- The authors thank the reviewer for this suggestion and agree this would be helpful for the reader’s context. We have added a sentence to the second paragraph of Discussion: “Though we present here data showing IgG concordance with antibodies against PfMSP119, a complete report of *P. falciparum* seroprevalence and spatial estimates will be forthcoming.”

**Reviewer #2:**

Line 59: change “populace” to “population” – make sure “population” used throughout text for consistency Line 66: change “test for antibodies” to “testing for antibodies” or “tests for antibodies”

Line 67: change “burden to” to “burden of”

Line 72: change “rely” to “relies”

Line 88: change “of years” to “for years”

Line 105: change “will only identify” to “only identify”

Line 119: change “helminth” to “helminths”

- All changes have been made as suggested above

Line 295 – 297: would be helpful to have an estimate of the area covered by the north and south regions, and the region as a whole (in km2 for example) to give context into how large each of these sub-areas are compared to the whole region.

- The authors are unsure what the reviewer is requesting in this comment. We have included a sentence at the beginning of this paragraph that states the total surface area of Haiti. The inclusion of km estimates presented in the text is not meant to be relative to the total geographical area of the country, and readers can note the relative sizes in Figure 3.

Line 336 – 342: when you specify the departments with more than 100 children sampled you should also state species to make it clear which you are talking about. For example: “For departments with more than one-hundred children sampled, P. vivax seroprevalence was highest in Sud-Est (3.3%)…”

- The authors agree with this clarification text and have added appropriate species names here.

Line 344: would be helpful to have a range alongside the average age of positive children.

- The authors thank the reviewer for the suggestion, but will remind the reviewer that sampling was biased towards 6-7y olds for this survey, and those ages encompass >97% of all enrolled children.

Line 348: use a symbol like a cross or star instead of a, or make “a” a superscript to make it clear it is a comment not a misplaced letter

- We have made this into a superscript to avoid confusion.

Line 388: remove “the” from “in the northern”

- Removed

Line 395: make sure “k” in K-function is capitalized

- Corrected

Line 395: could be good to remind the reader here how the K-functions are weighted

- The authors do not wish to reiterate in Results the information that’s included in Methods.

Line 431: make sure malaria spp is italicized throughout text (also unitalicized on line 528)

- This has been corrected.

Line 460: remove “which”

- This has been removed.

Line 521: remove “not”

- This has been removed

**Reviewer #3:**

In figure 3, I maintain that adjusting for sampling would be useful. the number of seropositive students per school isn't very informative if sampling isn't entirely uniform between schools and therefore I think the color coding of schools by seropositive students per school could be misleading. I believe this to be a rather minor concern that doesn't threaten overall conclusions.

- The authors thank the reviewer for this suggestion and assume the suggestion here is in reference to Figure 2 (not Fig 3 which displays spatial ellipses). The purpose of this Figure 2 is to provide a reader with a few pieces of information: the location of all schools sampled, relative number of children sampled in the Haitian departments, and a general snapshot of where the seropositives are being found. The information from this figure then flows into Figure 3 where spatial analyses confirms many of these areas of visually more positives are, in fact, significant by scanning statistics. As seroprevalence for both Pm and Pv is so low, and so many schools have zero seropositive children, the authors feel the current Figure 2 displays the intended information for the reader.

**Deputy editor:**

The authors have improved the paper.

I remained concerned about the implausibly large hotspot – it does not tally with the data in Figure 2. I think this is likely to be just a matter of changing the maximum size of the scan window to something justifiable. (It does not need to be justifiable on the grounds of interventions if they are not to be used, but should be justifiable for some reason other than that it was the default software setting).

- The authors agree with this comment and realize that the inclusion of this ellipse is unnecessary and ultimately an artifact of seropositive children in schools very far apart from each other. This Pv cluster has been removed from Figure 3 as well as Table 3 and any references to it throughout the paper removed as well.

I agree with Reviewer 2 (L404-430) who requested actual p-values rather than p<0.05 or p<0.01. Statistics has moved away from these cut-offs now and textbooks recommend using the exact p-value since they contain more information and also give a more accurate impression of the test that was carried out. Please use the exact p-values.

- We agree with this assessment and rationale and have provided the exact p-values in this paragraph.

The sentence added at the end of the Introduction about the rationale for the analysis does not seem accurate. An ‘analysis’ does not aim to provide a rationale for something, it only answers a specific question. Do you mean the aim of the ‘study’ is to provide a rationale for something else? It would make more sense if the aim of the study would be to summarize the spatial clusters of Pf and Pm in Haiti, and to demonstrate the use of IgG. Otherwise it does rather sound as if the aim of research is to justify research.

- The authors completely agree with this suggestion and have re-worded the final sentence of Introduction: “This study aims to utilize IgG data to determine spatial transmission patterns for the very low endemic malaria species of *P. malariae* and *P. vivax* in Haiti.”
