# Supplementary material for: Spatial cluster analysis of Plasmodium vivax and P. malariae exposure using serological data among Haitian school children sampled between 2014 and 2016
Source: PLoS Negl Trop Dis. 2022 Jan 5;16(1):e0010049. doi: 10.1371/journal.pntd.0010049 (PMC8765618; doi:10.1371/journal.pntd.0010049)
Supplement: Attachment — Submitted filename: Oviedo_etal_Resubmission3_Response to Editor.docx [file pntd.0010049.s007.docx]

Oviedo, et al, Response to Editor

The authors have adequately addressed all reviewer critiques, with one exception. Please address the following issue:

Line 269 "The north region included the departments Nord, Nord-Est, Nord-Ouest, Artibonite, and Centre. The southern region included the departments, Grand'Anse, Sud, Nippes, and Sud-Est."

Please give total land areas for these subregions. As you state in the previous rebuttal, you do already provide the total landmass of Haiti. However, it is useful for the reader to understand the two subareas that are analyzed, especially since one part of the nation is excluded (Oueste). This should be easy to calculate/estimate with the GIS software used. Without this it would be easy for a reader to think that the entire nation was included in this analysis. Also consider a very brief statement about why Oueste is excluded.

The authors thank the editor for this suggestion, and have added to Line 270 of Methods:

“The north region included the departments Nord, Nord-Est, Nord-Ouest, Artibonite, and Centre which provided a cumulative surface area of 14,315 km^2^. The southern region included the departments, Grand’Anse, Sud, Nippes, and Sud-Est which provided a cumulative surface area of 7,868 km^2^. The department of Ouest was excluded for clustering analyses as so few children were enrolled for collection of both *P. malariae* and *P. vivax* serology data.”
